# Early detection of gastric cancer *via* high-resolution terahertz imaging system

**DOI:** 10.3389/fbioe.2022.1052069

**Published:** 2022-12-14

**Authors:** Han Shi, Tenghui Li, Zhaoyang Liu, Junhua Zhao, Feng Qi

**Affiliations:** ^1^ Shenyang Institute of Automation, Chinese Academy of Sciences, Shenyang, China; ^2^ Department of Surgical Oncology and General Surgery, the First Hospital of China Medical University, Shenyang, China; ^3^ Key Laboratory of Precision Diagnosis and Treatment of Gastrointestinal Tumors (China Medical University), Ministry of Education, Shenyang, China; ^4^ Key Laboratory of Terahertz Imaging and Sensing, Liaoning Province, Shenyang, China

**Keywords:** terahertz, imaging, gastric cancer, H&E staining, high-resolution

## Abstract

Terahertz (THz) wave has demonstrated a good prospect in recent years, but the resolution is still one of the problems that restrict the application of THz technology in medical imaging. Paraffin-embedded samples are mostly used in THz medical imaging studies, which are thicker and significantly different from the current gold standard slice pathological examination in sample preparation. In addition, THz absorption in different layers of normal and cancerous tissues also remains to be further explored. In this study, we constructed a high-resolution THz imaging system to scan non-tumorous adjacent tissue slices and gastric cancer (GC) tissue slices. In this system, a THz quantum cascade laser emitted a pulsed 3 THz signal and the transmitted THz wave was received by a THz detector implemented in a 65 nm CMOS process. The slice thickness was only 20 μm, which was close to that of the medical pathology examination. We successfully found THz transmittance differences between different layers of normal gastric tissues based on THz images, and the resolution could reach 60 μm for the first time. The results indicated that submucosa had a lower THz transmittance than that of mucosa and muscular layer in non-tumorous adjacent tissue. However, in GC tissue, THz transmittance of mucosa and submucosa was similar, caused by the decreased transmittance of mucosa, where the cancer occurs. Therefore, we suppose that the similar terahertz transmittance between gastric mucosa and submucosa may indicate the appearance of cancerization. The images obtained from our THz imaging system were clearer than those observed with naked eyes, and can be directly compared with microscopic images. This is the first application of THz imaging technology to identify non-tumorous adjacent tissue and GC tissue based on the difference in THz wave absorption between different layers in the tissue. Our present work not only demonstrated the potential of THz imaging to promote early diagnosis of GC, but also suggested a new direction for the identification of normal and cancerous tissues by analyzing differences in THz transmittance between different layers of tissue.

## 1 Introduction

Gastric cancer (GC), as one of the most common cancer worldwide, is the world’s major healthcare burden (Joshi and Badgwell, 2021). In recent years, many studies on improving prognosis of patients with GC have been conducted (Alberts et al., 2003; Charalampakis et al., 2018; Salati et al., 2019), and 5-year survival rate can reach 90% in those at early stage (Song et al., 2017). However, the outcome of advanced GC is still poor (Digklia and Wagner, 2016). GC usually infiltrates from the superficial mucosa to the deeper tissue and is frequently diagnosed at advanced stage (Bray et al., 2018). Thus, it is important to improve the diagnostic method of GC.

At present, the imaging diagnosis of cancer mainly depends on computed tomography (CT) and magnetic resonance imaging (MRI). CT as a common technique used to assess tumor invasion, has a short scan time and can image both thorax and abdomen at the same time (Borggreve et al., 2019). MRI is significantly more sensitive to minor lesions and avoids radiation exposure (Brenner et al., 2014; Ohana et al., 2018). However, health concerns may lead to over-testing of patients (Jankauskaite et al., 2022). This phenomenon also occurs in low-income and middle-income countries (Albarqouni et al., 2022). It could be harm to patients and cause unnecessary financial costs. For example, the ionizing radiation from CT scans could cause DNA damage and increase the risk of cancer (Pearce et al., 2012). And the MRI has a high cost and relatively long acquisition times (Bor et al., 2016; Heidkamp et al., 2021). In addition, the sensitivity of CT and MRI in the detection of micro lesions of cancer still needs to be improved (Gai et al., 2021; Liu et al., 2021). As a new type of diagnostic technology, optical imaging has the characteristics of no ionizing radiation exposure and high spatial resolution in cancer diagnosis (Wang et al., 2018). Nevertheless, this technique often requires the use of additional specific probes or dyes, which limits its clinical application. The gold standard for cancer diagnosis is pathological examination. However, it has a long diagnosis period. In addition, compared with examinations based on H&E staining slices, the results of electromagnetic wave detection could be relatively objective. Therefore, there is an urgent need for a technique, which is non-invasive and high-accuracy for cancer diagnosis.

The discovery of new electromagnetic waves often leads to the development of new medical diagnostic and imaging tools (Xiang et al., 2020, Lou et al., 2022). Terahertz (THz) light is a class of electromagnetic waves that the frequency is in the range of 0.1–10 THz (Shumyatsky and Alfano, 2011). Unlike x-rays used in traditional examinations, THz light’s frequency is about one-million-time lower. Due to this feature, THz radiation’s low-energy photons, which range between 0.4 and 41 millielectronvolts (meV), have no hazard of ionizing radiation (Yang et al., 2016). THz imaging technology has made great achievements in biomedical imaging. It is quite important to extract information from THz images and a sound understanding of interactions between THz waves and tissues will contribute, including absorption, scattering, corresponding path loss *etc.* (Liu et al., 2019). Influence of the effects above can be investigated by MonteCarlo simulations, similar to the work in (Lin et al., 2022). THz imaging system can be divided into different systems according to their structures. Based on terahertz pulsed imaging (TPI) system, researchers have been able to observe the boundary between cancerous and normal tissue in laryngeal cancer, breast cancer, and mouse brain glioma (Oh et al., 2014; Chavez et al., 2018; Vohra et al., 2020; Ke et al., 2022). By using continuous-wave terahertz (CW THz) transmission or reflection scanning imaging system, the difference of THz images between cancerous and normal tissues in colon cancer and mouse brain glioma was detected respectively (Wahaia et al., 2016; Wu et al., 2019). With CW THz near-field microscopy imaging system, researchers successively distinguished cancerous tissues from normal tissues in stomach and colon (Chen et al., 2015; Chen et al., 2022). Based on attenuated total reflection (ATR) imaging system, THz absorption difference between mouse brain glioma and normal mouse brain tissue was observed (Wu et al., 2022). However, previous studies usually focused on the difference in terahertz absorption between cancerous and normal tissues. Further exploration of THz absorption by different fine structures of tissue will promote the application of THz technology in clinical diagnosis. The resolution of THz imaging needs to be improved as well. The use of thinner slices instead of paraffin-embedded samples could also facilitate the observation of fine structure of the tissue. In addition, analysis of normal tissue with THz imaging technology could help explore the difference between normal and cancerous tissue, which may provide a more reliable basis for clinical diagnosis, and study the transformation process of normal tissue into cancerous tissue from the perspective of physics.

In this study, a high-resolution THz imaging system was designed. At 3 THz, the resolution of the scanned image is 60 μm, which is clearer than observation with naked eyes and can be directly compared with microscopic images. With this high-resolution imaging system, we then investigated the THz transmittance of different layers of normal and cancerous tissue by detecting slices of non-tumorous adjacent specimens and GC specimens. The variation of THz transmittance difference between different layers of normal and cancerous tissue reveals the potential of THz in assisting early diagnosis of GC.

## 2 Materials and methods

### 2.1 Experimental system setup introduction

**FIGURE 1:**
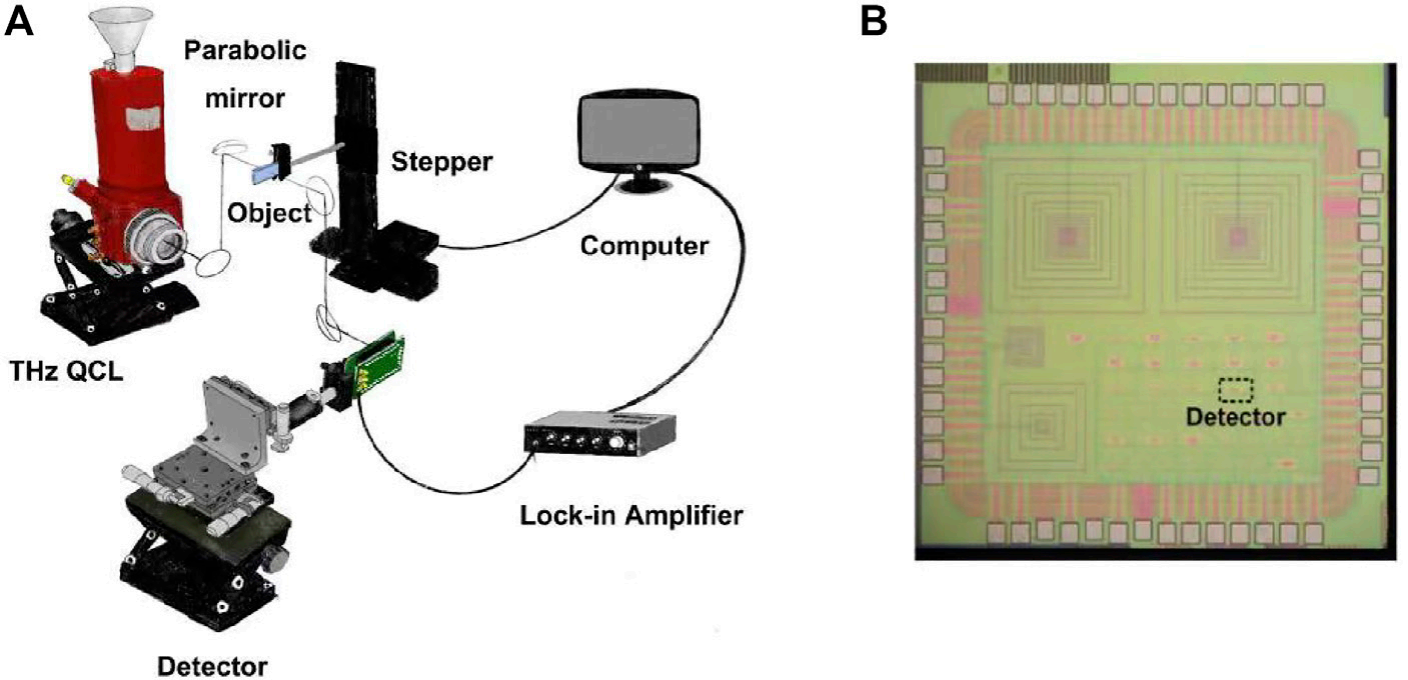
**(A)** Block diagram of the THz imaging system and **(B)** die photo of the THz detector.

The THz imaging system is shown in Figure 1A. A THz quantum cascade laser (QCL) emits a pulsed 3 THz signal with a repetition rate of 5 kHz and a duty cycle of 1%. Two parabolic mirrors are used to focus the THz radiation on an object, which is fixed on a two-dimensional stepper to realize scanning image. The transmitted THz radiation is collimated and focused on a THz detector by two other parabolic mirrors. The output signal of the detector is captured by a lock-in amplifier and then collected by a computer. The detector is shown in Figure 1B, which is a 3 THz detector implemented in a 65 nm CMOS process. The resolution of the imaging system is about 60 μm.

### 2.2 Human tissue samples

The samples were obtained from patients undergoing surgical resection of GC at the First Hospital of China Medical University during 2016. After surgery, the fresh specimens were fixed in formalin, routinely processed, and embedded in paraffin, then stored as tissue blocks. The samples were sectioned for H&E staining (5 μm) and THz imaging (20 μm), the surrounding paraffin was melted away for THz imaging, and only the relative flat tissues were selected for experiments.

### 2.3 Ethical approval

This study followed the principles outlined in the Declaration of Helsinki. Required specimens were collected through informed patient consent and in accordance with institutional ethical guidelines. The study protocol was reviewed and approved by the Research Ethics Committee of China Medical University (Shenyang, China).

### 2.4 H&E staining

The tissue slices (5 μm) were immersed in xylene for deparaffinization and rehydrated in graded ethanol solutions for staining in hematoxylin solution (5 min). They were then dipped in 1% hydrochloric acid ethanol, washed in distilled water, and stained in eosin solution (3 min). Finally, graded alcohol solutions were applied for dehydration and subsequent immersion in xylene. The stained slices were examined by a Leica DM4000B microscope (Leica, Wetzlar, Germany).

### 2.5 Relative transmittance determination

Combined with histopathological examination, the boundaries of mucosa, submucosa and muscular layer were outlined with dashed blue lines. For each layer of tissue sample in the images, we randomly select nine points in the region with uniform color to calculate the average transmittance as the transmittance of this layer. To quantify transmissivity, we calculated the relative transmittance (RT) to compare the transmittance of different layers in each of the collected samples. RT between mucosa and submucosa was defined as RT = (T_m_-T_s_)/T_m_, where T_m_ was averaged transmittance of labeled mucosa area in the sample and T_s_ was averaged transmittance of labeled submucosa area in the same specimen. This was to avoid the influence of tissue variation between different samples on the overall transmittance.

### 2.6 Statistical analysis

All data comparisons were based on Student’s *t*-test and were computed *via* standard software (SPSS v25.0; SPSS Inc., Chicago, IL, United States), by setting significance at *p* < 0.05.

## 3 Results

### 3.1 Collected samples

Overall, eight collected tissue samples were tested during the study, which included three non-tumorous adjacent tissues (NATs) (N1, N2, N3) and five primary GC lesions (T1, T2, T3, T4, T5). N1 and T1 were from a 42-year-old woman (P1); N2 and T2 were from a 52-year-old man (P2); T3 was from a 76-year-old man (P3); T4 was from a 64-year-old woman (P4); N3 and T5 were from a 55-year-old woman (P5). Patient characteristics are shown in Table 1. The general shapes of all 8 samples can be distinguished in THz image.

**TABLE 1:** Basic information of patients included.

Patient	Sex	Age	Lesions position	T stage*	Infiltration depth	Histological type	Sample size (mm)
P1	female	42	Gastric antrum	T_3_	subserosa	Mucinous adenocarcinoma	14 × 28×0.02
P2	male	52	Gastric antrum	T_4_	serosa	Adenocarcinoma	13 × 22×0.02
P3	male	76	Gastric antrum	T_3_	subserosa	Papillary tubular adenocarcinoma	70 × 75×0.02
P4	female	64	Gastric antrum	T_2_	muscularis propria	Adenocarcinoma	16 × 15×0.02
P5	female	55	Gastric body	T_1_	mucosa	Adenocarcinoma	12 × 18×0.02

* The histological depth of tumor invasion.

### 3.2 Difference of THz transmittance between different layers of normal gastric tissue

**FIGURE 2:**
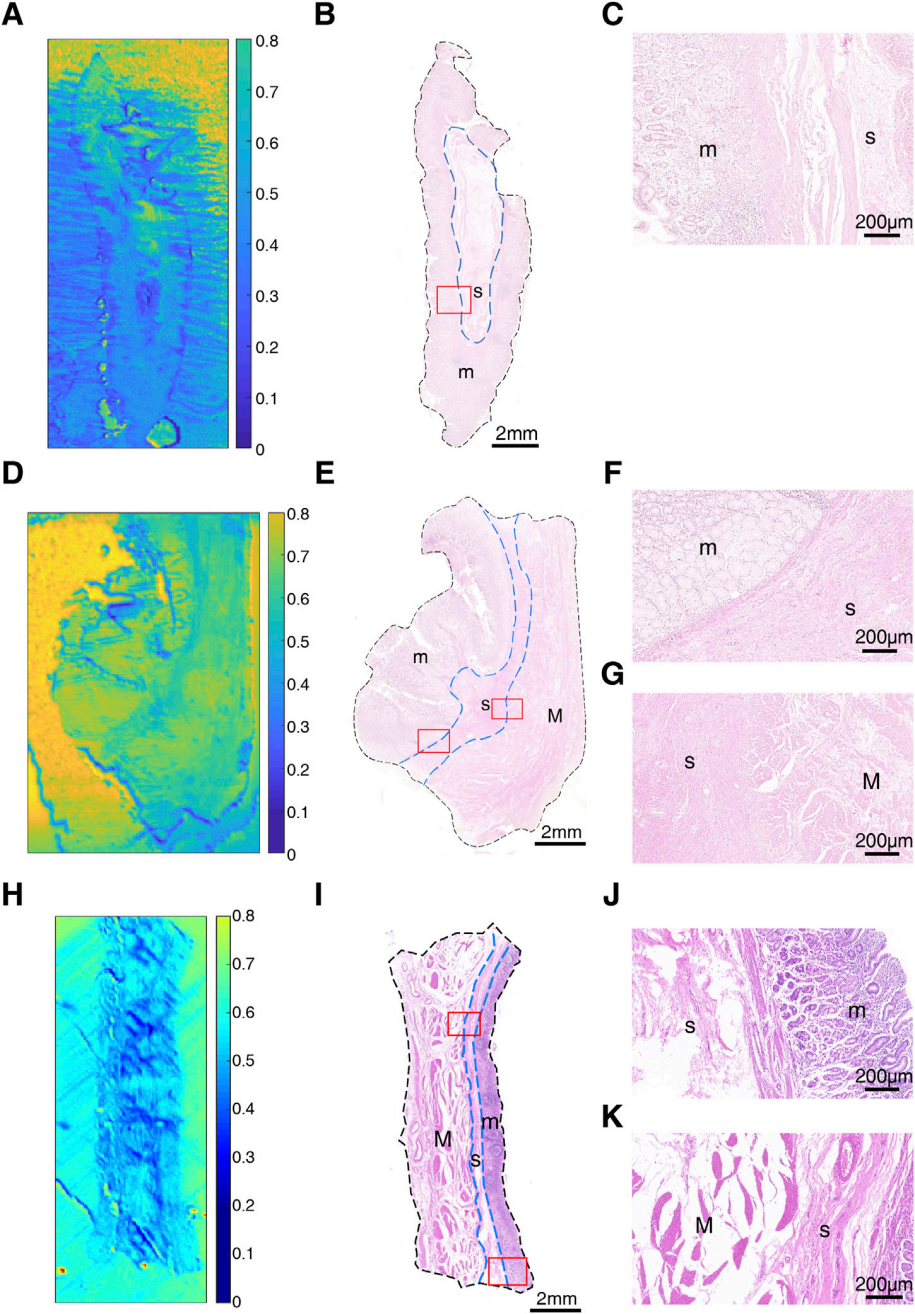
Images of non-tumorous adjacent tissue: **(A)** THz image, **(B)** H&E-stained slice, and **(C)** H&E-stained image corresponding to the region indicated by the red square in **(B)** of N1; **(D)** THz image, **(E)** H&E-stained slice, and **(F,G)** H&E-stained images corresponding to the region indicated by the red square in **(E)** of N2; **(H)** THz image, **(I)** H&E-stained slice, and **(J,K)** H&E-stained images corresponding to the region indicated by the red square in **(I)** of N3. In H&E-stained images, “m” indicates “mucosa”, “s” indicates “submucosa” and “M” indicates “muscular layer”. In **(B,E,I)**, the boundaries of mucosa, submucosa and muscular layer are outlined with dashed blue lines. **(C,F,J)** are boundaries between mucosa and submucosa. **(G,K)** are the boundaries between submucosa and muscular layer.

To explore the transmittance characteristics of normal gastric tissue, we measured and acquired THz images of non-tumorous adjacent tissue N1, N2 and N3 from three GC patients (P1, P2 and P5) (Figure 2). It is shown that THz images had a relative high resolution, corresponding to the position and shape of different tissue layers (mucosa, submucosa, and muscular layer) in H&E examination of these three samples. Figures 2A,D,H were THz images of collected NAT. The boundaries of mucosa, submucosa and muscular layer are outlined with dashed blue lines in H&E-stained slice (Figures 2B,E,I). The magnified H&E-stained images of boundaries between different layers of tissue were also presented in Figures 2C,F,G,J,K. N1 was collected from the NAT from a 42-year-old woman P1 (Figures 2A–C). The T_m_ and T_s_ in this sample were 0.745 (±0.057) and 0.485 (±0.077) respectively. The RT of N1 was 0.349. N2 was the slide of the NAT from a 52-year-old man P2 (Figures 2D–G). The T_m_, T_s_ and T_M_ in this sample were 0.722 (±0.014), 0.574 (±0.048) and 0.690 (±0.031), respectively. The RT of N2 was calculated to be 0.205. N3 was the slide of the NAT from a 55-year-old woman P5 (Figures 2H,K). The T_m_, T_s_ and T_M_ in this sample were 0.623 (±0.030), 0.519 (±0.047) and 0.599 (±0.042), respectively. The RT of N3 was calculated to be 0.166. RT was defined as the difference in the transmittance of mucosa and submucosa divided by mucosa transmittance. The results showed that the THz transmittance was different between mucosa and submucosa.

**FIGURE 3:**
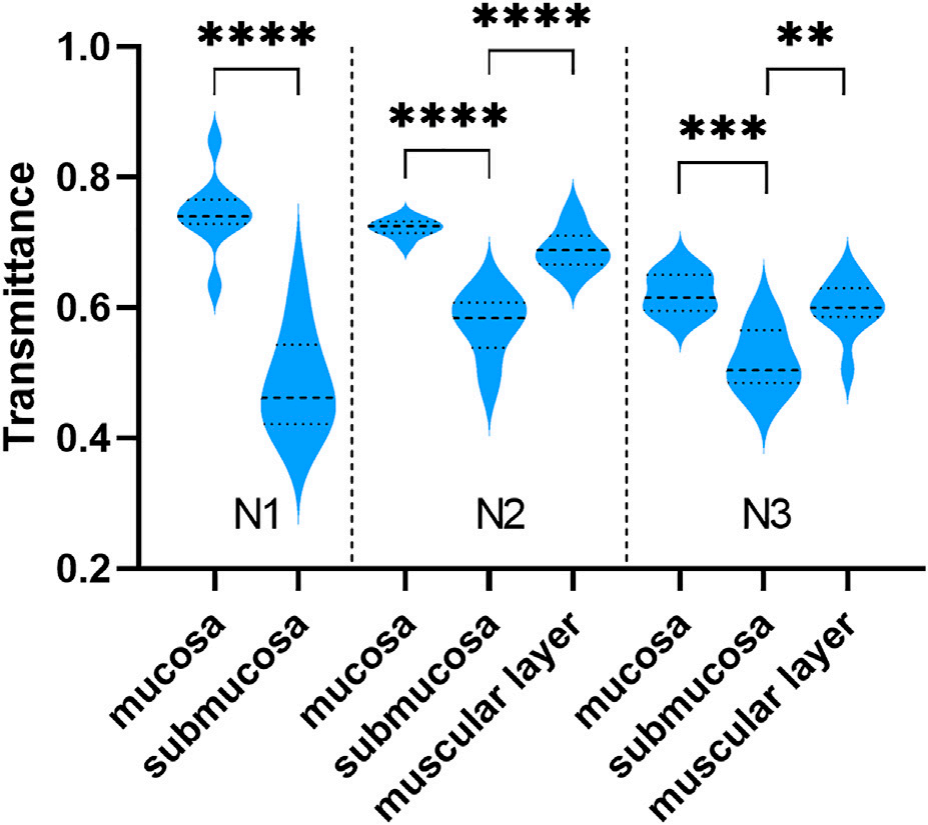
The transmittance of different layers in sample N1, N2, and N3. The transmittance of submucosa was lower than that of mucosa and muscular layer. ****: *p* < 0.0001, ***: *p* < 0.001, **: *p* < 0.01.

Although THz images could not identify tissue morphology of samples alone, it was able to distinguish the region of mucosa, submucosa, and muscular layer in normal gastric tissue through intense color contrast. As shown in Figure 3, the transmittance of submucosa is significantly lower than mucosa in all three samples. Besides, submucosa of sample N2 and N3 also presented a lower transmittance than muscular layer. The THz images of Figure 2 also clearly showed that THz transmittance of submucosa is lower than that of mucosa and muscular layer. That is, the submucosa absorbs more THz waves than mucosa and muscular layer in normal gastric tissue.

### 3.3 Variation of difference of THz transmittance between different layers of GC tissue

**FIGURE 4:**
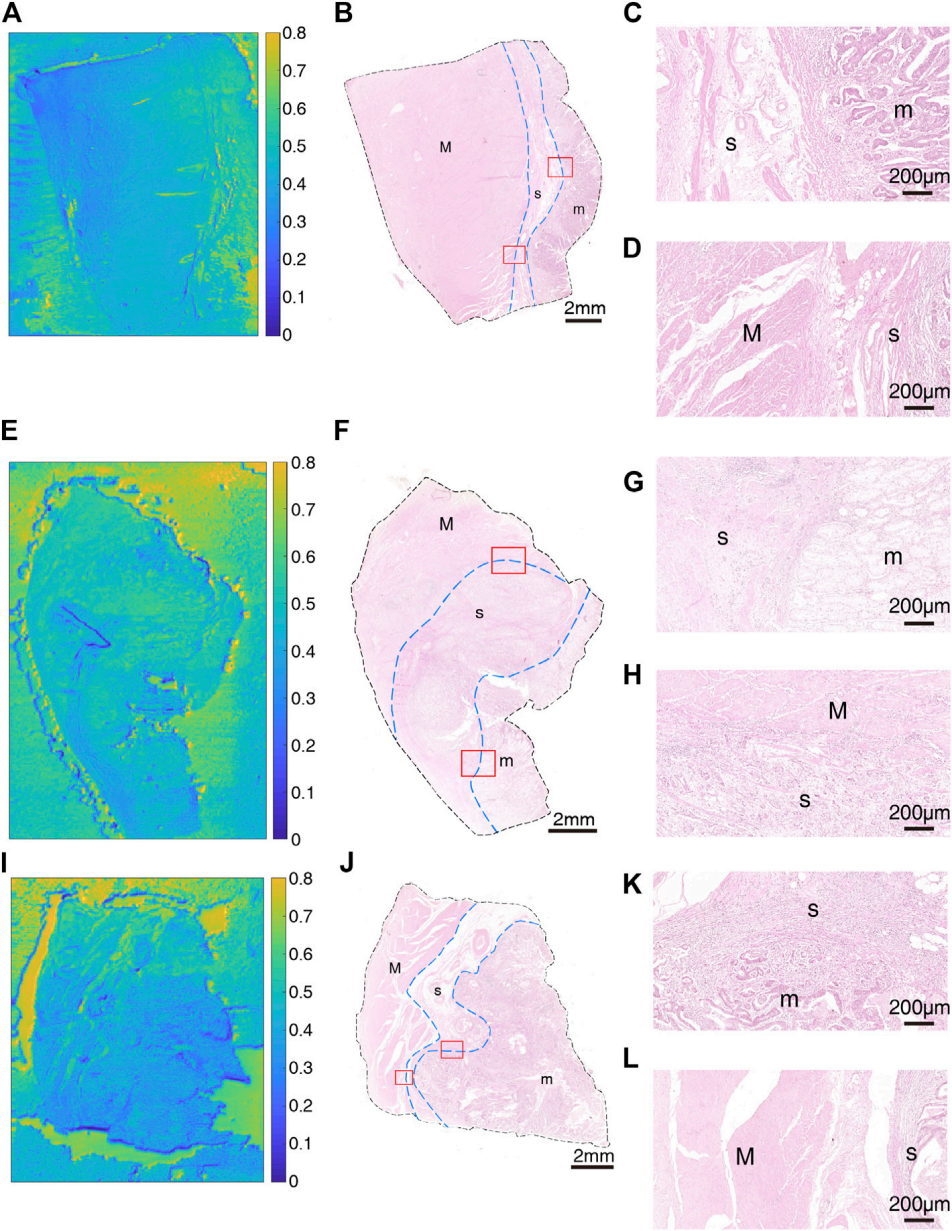
Images of GC sample: **(A)** THz image, **(B)** H&E-stained slice, and **(C,D)** H&E-stained images corresponding to the region indicated by the red square in **(B)** of T1; **(E)** THz image, **(F)** H&E-stained slice, and **(G,H)** H&E-stained images corresponding to the region indicated by the red square in **(F)** of T2; **(I)** THz image, **(J)** H&E-stained slice, and **(K,L)** H&E-stained images corresponding to the region indicated by the red square in **(J)** of T3. In H&E-stained images, “m” indicates “mucosa”, “s” indicates “submucosa” and “M” indicates “muscular layer”. In images **(B,F,J)**, the boundaries of mucosa, submucosa, and muscular layer are outlined with dashed blue lines. **(C,G,K)** are boundaries between mucosa and submucosa. **(D,H,L)** are boundaries between submucosa and muscular layer.

**FIGURE 5:**
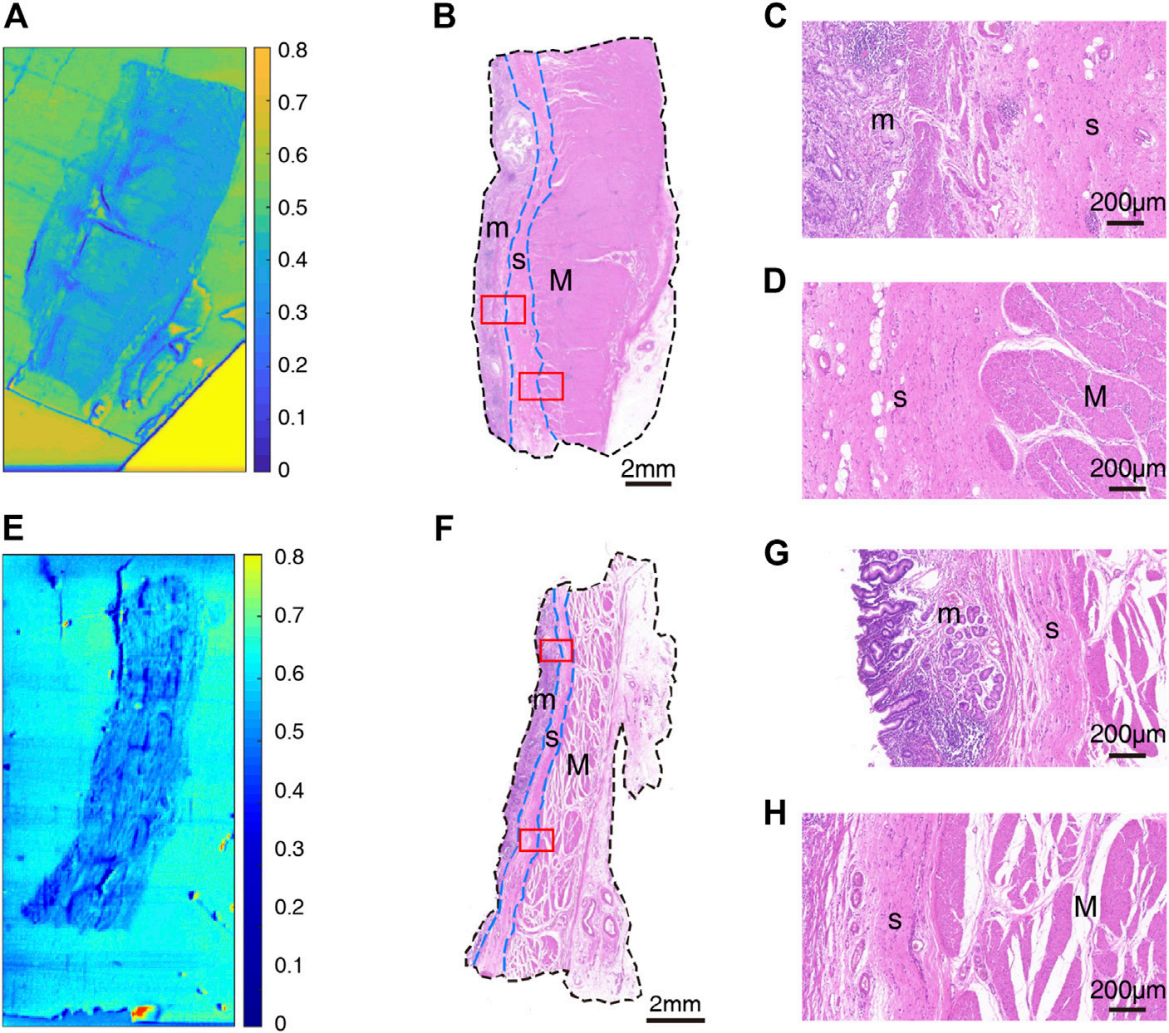
Images of GC sample: **(A)** THz image, **(B)** H&E-stained slice, and **(C,D)** H&E-stained images corresponding to the region indicated by the red square in **(B)** of T4; **(E)** THz image, **(F)** H&E-stained slice, and **(G,H)** H&E-stained images corresponding to the region indicated by the red square in **(F)** of T5. In H&E-stained images, “m” indicates “mucosa”, “s” indicates “submucosa” and “M” indicates “muscular layer”. In images **(B,F)**, the boundaries of mucosa, submucosa, and muscular layer are outlined with dashed blue lines. **(C,G)** are boundaries between mucosa and submucosa. **(D,H)** are boundaries between submucosa and muscular layer.

After that, we obtained THz images of primary cancer lesions T1, T2, T3, T4 and T5 from five GC patients (P1, P2, P3, P4, and P5) to explore whether there were changes in transmittance difference among different layers of GC tissue. Figures 4A,E,I and Figures 5A,E were THz images of collected GC tissues. The boundaries of mucosa, submucosa and muscular layer are outlined with dashed blue lines in H&E-stained slices (Figures 4B,F,J and Figures 5B,F). The magnified H&E-stained images of boundaries between different layers of tissue were also presented in Figures 4C,D,G,H,K,L, and Figures 5C,D,G,H. T1 was collected from the GC tissue from a 42-year-old woman P1 (Figures 4A–D). The T_m_, T_s_ in this sample were 0.520 (±0.045) and 0.483 (±0.050) respectively. The RT was calculated to be 0.071. T2 was from the GC tissue from a 52-year-old man P2 (Figures 4E–H). The T_m_, T_s_ in this sample were 0.509 (±0.019) and 0.530 (±0.021) respectively. The calculated RT was -0.041. T3 was collected from the GC tissue from a 76-year-old man P3 (Figures 4I–L). The T_m_, T_s_ in this sample were 0.382 (±0.027) and 0.411 (±0.032) respectively. The RT was calculated to be −0.076. T4 was from the GC tissue from a 64-year-old woman P4 (Figures 5A–D). The T_m_, T_s_ in this sample were 0.439 (±0.023) and 0.408 (±0.045) respectively. The calculated RT was 0.069. T5 was collected from the GC tissue from a 55-year-old woman P3 (Figures 5E–H). The T_m_, T_s_ in this sample were 0.533 (±0.051) and 0.524 (±0.048) respectively. The RT was calculated to be 0.017. RT was defined as the difference in the transmittance of mucosa and submucosa divided by mucosa transmittance. The results revealed that the THz transmittance between mucosa and submucosa was similar.

**FIGURE 6:**
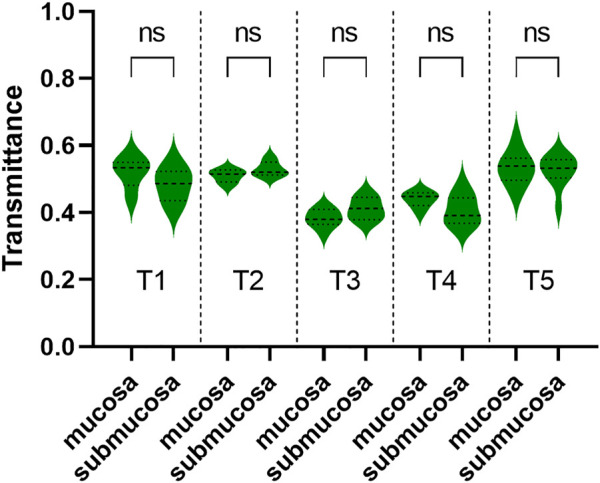
The transmittance of mucosa and submucosa in T1, T2, T3, T4, and T5. The transmittance of these two layers was similar in each of the three samples. ns: not significant.

**FIGURE 7:**
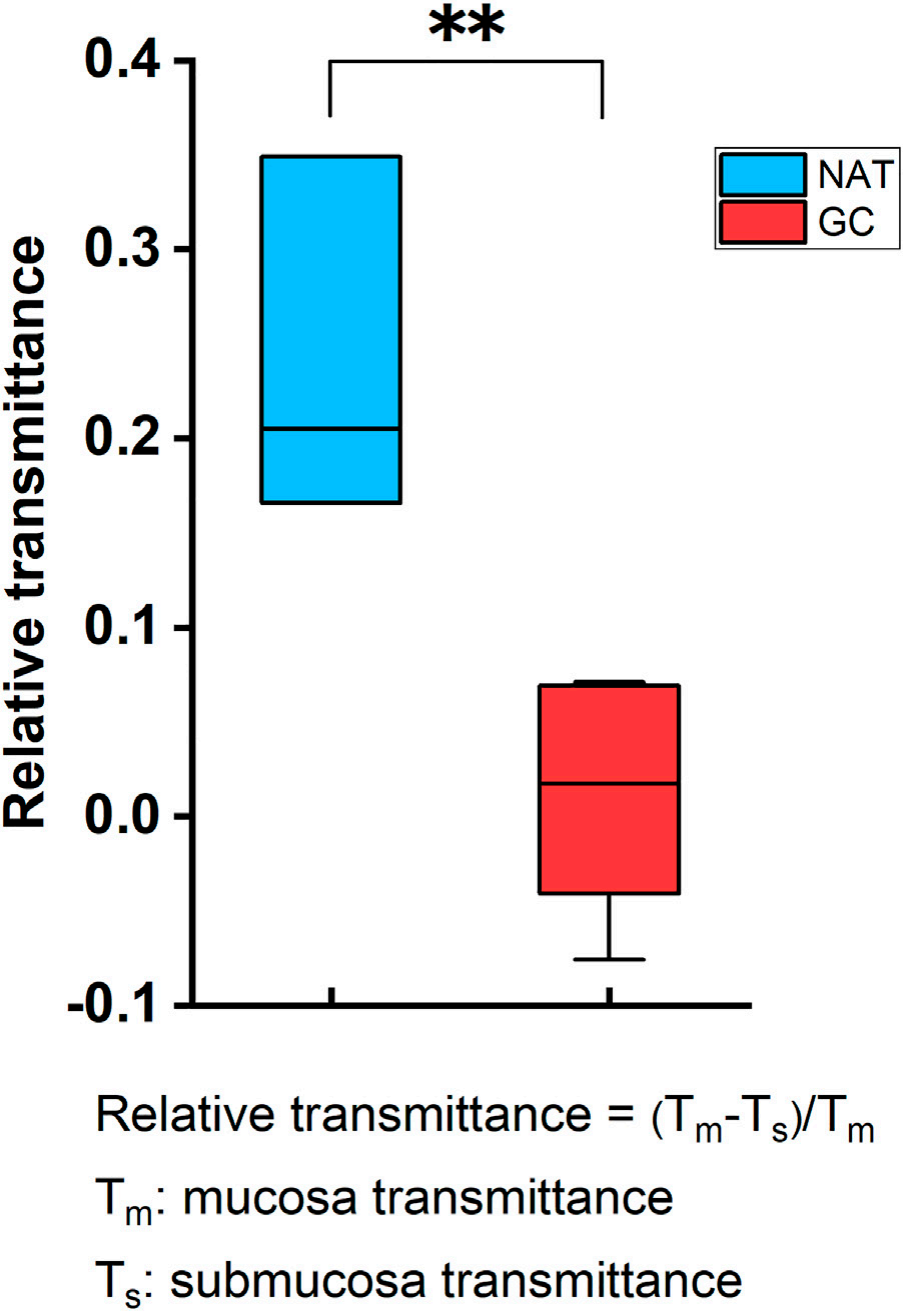
The difference of RT between NAT and GC tissue. The transmittance difference between mucosa and submucosa in NAT was significantly higher than that in GC tissue. RT between submucosa and mucosa was defined as RT = (T_m_-T_s_)/T_m_, where T_m_ was averaged transmittance of mucosa and T_s_ was averaged transmittance of submucosa. **: *p* < 0.01.

Samples’ THz images in Figures 4, 5 also showed that the THz transmittance of mucosa is similar to that of submucosa in sample T1, T2, T3, T4 and T5. Similarly, as Figure 6 presented, there was no significant difference in transmittance between mucosa and submucosa in all five samples. Although the boundaries of mucosa and submucosa in H&E-stained images were still relatively visible, it was difficult to accurately identify the areas of different tissue by color contrast in THz images. This is due to the fact that GC mainly rises from mucosa, which leads to a decrease in THz transmission, so the THz absorption capacity of mucosa is similar to that of submucosa. Based on the findings above, we analyzed RT of three NAT samples and five GC tissue samples to investigate the variation of RT between NAT and GC tissue. The results in Figure 7 revealed a statistical difference of RT between normal and cancerous gastric tissue (*p* = 0.006). The results suggested that our THz imaging technology had the potential to detect cancers by measuring the transmittance difference between different layers of tissue.

## 4 Discussion

In our study, we designed a high-resolution THz imaging system to scan gastric tissue slides. There was a novel finding that the THz transmittance of mucosa, submucosa and muscular layer were different in normal gastric tissue. The transmittance of submucosa was relatively low, while the transmittance of mucosa and muscular layer were relatively high. It was also found that the transmittance between mucosa and submucosa in GC tissue was similar. We believe this is due to the fact that cancerous tissue mainly arises from the mucosa, resulting in a decrease in THz transmittance.

Considerable progress has been made in the research on the biological application of electromagnetic wave (Zhang et al., 2021; Li et al., 2021). A growing number of studies have shown that THz is a useful approach for identifying cancers because malignant tumors usually contain more water and can absorb large amounts of THz radiation (Reid et al., 2011; Wahaia et al., 2011; Yamaguchi et al., 2016; Wu et al., 2022). The difference in THz transmittance among layers of normal gastric tissue may be due to water content differences as well. CW THz transmission scanning imaging system has shown great potential in the diagnosis of non-melanoma skin cancer, breast cancer and colon cancer (Joseph et al., 2011; Bowman et al., 2016; Wahaia et al., 2016). In addition, the CW THz near-field microscopy imaging system has made some achievements in the identification of cancerous and normal gastric tissues. Nevertheless, these studies have not yet enabled high resolution imaging of the fine structure of tissues. Moreover, current studies on the use of THz imaging to diagnose cancer are usually based on the difference of THz parameters between normal and cancerous tissue (Chen et al., 2022; Ke et al., 2022; Wu et al., 2022). Our study provided a new idea for THz diagnosis of GC. By calculating the difference in transmittance between mucosa and submucosa, the existence of cancerization in tissue sample could be inferred. In addition, we obtained THz images with a resolution of up to 60 μm for the first time, while the thickness of the scanned slice was only 20 μm. Thinner slices are also conducive to further observe of the fine structure of the tissue. Meanwhile, the slices’ thickness was close to that of the slices used in clinical pathology examination, facilitating direct comparison of THz images with microscopic images. Our THz transmission imaging system is expected to save time and workload on GC pathology examinations and provide convenience for pathologists.

In the GC tissue we collected, the THz transmittance of the muscular layer was similar to that of the submucosa in some samples (T2, T3, T4, and T5), while different in the other sample (T1). This may be related to different degrees of invasion of cancerous tissue. Our study could assist in the diagnosis of GC by comparing the transmittance difference between mucosa and submucosa. Future studies could further focus on inferring the invasion depth of cancerous tissue by THz imaging to distinguish between early and advanced GC.

In conclusion, we proposed a high-resolution THz imaging system to scan normal and cancerous gastric tissue slides. The resolution is up to 60 μm. With this imaging system, we explored the THz transmittance characteristics of different layers of gastric wall tissue and identify them in THz images for the first time. Moreover, it is impressive that the THz transmittance of submucosa is lower than that of mucosa in NAT slides, but the THz transmittance of submucosa is similar to that of mucosa in GC tissue slides. We hypothesize that similar terahertz transmittance between gastric mucosa and submucosa may be an indication of carcinogenesis. We reported this phenomenon hoping to provide readers with more information. In future studies, we will explore this phenomenon in depth to provide more evidence.

## Data Availability

The raw data supporting the conclusion of this article will be made available by the authors, without undue reservation.
